# Knockout of filamin A in KGN granulosa tumor cells impairs proliferation, cell cycle progression, migration, and cytoskeletal organization under mechanical stress

**DOI:** 10.1186/s40659-026-00686-x

**Published:** 2026-05-20

**Authors:** Yuhao Jiang, Karolina Magdalena Caban, Mirko Peitzsch, Carola Herrmann, Doris Mayr, Jan Bernd Stöckl, Thomas Fröhlich, Artur Mayerhofer, Annette Müller-Taubenberger, Harald Welter

**Affiliations:** 1 Cell Biology (Anatomy III), Faculty of Medicine, Biomedical Center (BMC), Ludwig Maximilian University, 82152 Munich, Planegg-Martinsried, Germany; 2 Laboratory for Functional Genome Analysis (LAFUGA), Gene Center, Ludwig Maximilian University, Munich, Germany; 3 Institute of Clinical Chemistry and Laboratory Medicine, University Hospital and Faculty of Medicine Carl Gustav Carus, Technical University of Dresden, Dresden, Germany; 4 Institute of Pathology, University Hospital, Ludwig Maximilian University, Munich, Germany

**Keywords:** Ovarian cancer cells, Granulosa cells, Filamin, CRISPR/Cas9, Shear stress, Steroids

## Abstract

**Background:**

Filamin A (FLNA) is an actin-binding protein that regulates mechanosensitivity and functions as an intracellular signaling scaffold in various cell types. It has also been implicated in tumor growth. We recently reported FLNA expression in human ovarian granulosa cells and in KGN cells, a granulosa cell tumor (GCT) line.

**Results:**

Immunohistochemistry analysis of 51 GCT samples revealed heterogeneous FLNA expression, with approximately 20% showing weak, 18% strong, and the majority moderate expression. We therefore conducted functional studies in KGN cells using CRISPR/Cas9 gene editing. A proteomic approach revealed marked changes in protein abundance upon *FLNA* depletion: proteins with increased abundance were predominantly related to adhesion, cytoskeletal organization, regulation of cell shape, and lipid metabolic process, whereas those with decreased abundance were associated with DNA replication, cell division, and cell cycle regulation. FLNA-knockout cells showed enlarged cell sizes, reduced proliferation, and slightly affected steroidogenesis. Disruption of FLNA further reduced migration velocity, altered actin cytoskeletal alignment under flow, and modified expression of genes involved in cytoskeletal architecture, adhesion, and mechanosensing under shear stress.

**Conclusions:**

Our results identify crucial roles of FLNA in shaping the cellular architecture, motility, and proliferation of KGN cells. Consequently, alterations in FLNA expression may influence intracellular signaling, and responsiveness to mechanical cues in both physiological and pathological contexts.

**Supplementary Information:**

The online version contains supplementary material available at 10.1186/s40659-026-00686-x.

## Background

Filamin A (FLNA) is one of the most studied actin-crosslinking proteins of recent decades and known to interact with hundreds of signalling molecules in response to various stimuli. As such, it influences fundamental cellular functions including shape, migration, adhesion, mechanosensing and signalling, but also differentiation and morphogenesis in many tissues and organs [1, 2].

Yet, in the human reproductive system, neither the presence, nor the function of FLNA have been thoroughly investigated, with few exceptions. Previously, we have identified FLNA in germ cells and peritubular cells of the human testis [3]. CRISPR/Cas9-based depletion of FLNA indicated a role in maintaining the cytoskeletal structure, the regulation of cell migration and an influence on stem cell characteristics in human TCam-2 seminoma cells [3].

More recently, we described the wide-spread expression of FLNA protein in human and nonhuman primate ovaries *in situ*, with pronounced localization in follicular granulosa and luteal cells as well as oocytes [4]. Using two established cellular models, human follicular granulosa cells (GC), derived from *in vitro* fertilization (IVF)-patients, and the human granulosa-like tumor cell line KGN, we provided first insights into its cellular localization and regulation. Furthermore, FLNA immunoprecipitation, followed by mass spectrometry, revealed a distinguished proteome pattern of these cells. These findings suggested that FLNA may be implicated in the regulation of GC function and hence may contribute to ovarian (patho-)physiology. In primary GC, in-depth studies into potential roles of FLNA are not readily possible and therefore we made use of the widely used cellular model, KGN cells. KGN cells are derived from an adult-type granulosa cell tumor (GCT) and display a steroidogenic profile similar to the one of normal GC. These rare yet potentially malignant tumors bear a Forkhead Box L2 missense mutation [5–7], which attribute to 2–5% of all ovarian cancers [8, 9]. Surgery remains the primary treatment, followed by chemotherapy, but often recurrence even years and decades after diagnosis is reported. Prognostic markers are actively being investigated, and current research is focused on identifying further key molecules with aberrant expression levels to predict the progression of GCTs [10].

FLNA may be such a candidate protein, at least in epithelial ovarian cancer. Zeng et al. identified FLNA as a potential biomarker for high-grade and low-grade serous carcinoma with a potential value for prognosis and treatment [11]. However, its role in oncological diseases is still controversial as current research suggests roles for *FLNA*, either as a potential oncogene, or as a gene that impedes tumor [2, 12, 13]. While reduced FLNA expression has been reported in bladder, prostate and colorectal cancers, in cases of melanoma, glioblastoma and breast cancer overexpression of FLNA has been observed [14].

To elucidate the (patho-)physiological significance of FLNA for GCT, we evaluated its expression in 51 human ovarian GCT samples and subsequently performed in depth-functional studies in KGN cells, using CRISPR/Cas9-based gene editing. FLNA-knockout induced pronounced phenotypic changes in KGN cells, including enlarged cell size, reduced proliferation and motility, increased steroid production, compromised cytoskeletal orientation and altered transcription of FLNA-associated genes under mechanical stress. The modifications in the proteome further supported substantial changes in key biological processes including adhesion, cytoskeletal organization and lipid metabolism, proliferation, and cell cycle regulation.

## Methods

### Human granulosa cell tumor (GCT) and tissue microarrays (TMAs)

Total RNA extracted from GCT samples of four patients (ages 41, 53, 66, and 31) was used for qPCR analysis, as previously described [15]. The use of these samples was agreed by all patients to the scientific use and approved by the ethics committee of the LMU (project 390-15). Additionally, archival TMAs comprising 51 GCT samples were utilized for immunohistochemical staining, as reported [16], with ethical approval from the LMU ethics committee (projects 20-697 & 20-895).

### Immunohistochemistry and evaluation of TMAs

The expression of FLNA protein using immunohistochemistry was assessed in TMAs of 51 samples of GCT tissues by the avidin–biotin-peroxidase technique [17], with each tissue double spotted on the slide. After the immunohistochemical procedure, sections were counterstained with hematoxylin. Negative controls consisted of mouse IgG (2 µg/mL, #I-5381 Sigma-Aldrich, Merck, Germany) instead of the primary antibody, or of omission of the first antibody.

TMAs were analyzed in a blinded manner by two independent investigators, and divergent results were re-evaluated. Due to the fact that all samples of GCTs spotted to TMA slides were determined to be FLNA-immunoreactive and showed exclusively cytoplasmic staining, a simplified Remmele and Stegner score [18] was used for evaluation: only the intensity of the immunohistochemical staining of GCTs was rated as negative to weak (negative = comparable to IgG control), moderate or strong. The analysis was performed at 10× and 40× objective magnification for each of the spotted tissues using a Zeiss Axioplan microscope (Carl Zeiss Microscopy, Germany) equipped with a Jenoptik camera (Jenoptik, Germany).

### Cell culture of KGN cells

Culture procedures of the patented KGN cell line originated from a GCT [5] (source: RIKEN BioResource Center, Japan) were reported previously in detail [15, 19]. In brief, KGN cells, in this study designated as wildtype (WT) cells (between passage numbers 20 to 40), were cultured in DMEM/F12 supplemented with 10% fetal calf serum (FCS), 0.1 µg/mL Normocin (InvivoGen Europe, France), and incubated at 37 °C with 5% CO_2_ until experimental use.

### Live cell imaging and nuclear staining

A total of 9 × 10^4^ WT or 5 × 10^4^ KO cells were seeded into a μ-Dish (35 mm, ibiTreat, ibidi, Germany). Due to differences in cell size, different seeding numbers for each cell type were chosen to ensure comparable cell confluency at the start of the experiment. After attachment of cells, NucBlue™ Live ReadyProbes™ Reagent (Thermo Fisher Scientific, USA) was added to the dish (two drops per milliliter of medium) and incubated for 20 min at room temperature (RT). The cells were washed with 1 mL PBS, then 2 mL DMEM/F12 medium containing 10% FCS and 0.1 µg/mL Normocin was added. Images were acquired using a Zeiss Z1 Axio Observer microscope equipped with a 40×/1.4 NA PlanApo oil immersion objective (Carl Zeiss Microscopy, Germany) and ZEN acquisition software (ZEN blue 2.6).

### CRISPR/Cas9 plasmid, design of sgRNAs and cloning

CRISPR/Cas9-based deletion of *FLNA* in KGN cells was performed with the same sgRNAs as used in the previously established protocol [3] with minor modifications as follows: sgRNAs were cloned into the eSpCas9(BB)-2A-Puro (PX459 V2.0; plasmid #62,988, Addgene, USA) with puromycin as selection marker. 12 to 14 days after transfection, puromycin resisted clones were detected and clonally expanded in 96-well plates. Four clones were selected for further experiments. To evaluate effective in-frame deletions in the *FLNA* gene of these clones upon Cas9 cleavage, genomic DNA was isolated and regions close to sgRNA targeted sites were amplified by polymerase chain reaction (PCR) and finally sequenced to further confirm gene editing events.

### Genotyping PCR

Genomic DNA from 5 × 10^5^ WT and FLNA-knockout (KO) cells was extracted using the Wizard® SV Genomic DNA Purification System (#A2360, Promega, USA) according to the manufacturer’s instructions. Genotyping was performed by PCR to amplify a 1031 bp fragment of the target locus (primers listed in Suppl. Table 1). Each 25 µL reaction contained 2 µL genomic DNA, 1 μM of each primer, 5 μL of 5 × Green GoTaq-Reaction-Buffer (#M791, Promega, USA), 0.125 µL GoTaq DNA-Polymerase (#M830, 5 U/µL; Promega, USA) and nuclease-free water. PCR cycling conditions were: 95 °C for 2.5 min; 35 cycles of 95 °C for 45 s; 59 °C for 30 s; 72 °C for 45 s; and a final extension at 72 °C for 5 min (Eppendorf® Mastercycler® Nexus gradient, Germany).

PCR products were separated on a 1.5% agarose gel containing Midori Green Advance DNA Stain (#MG04, 1:20,000, Nippon Genetics Europe, Germany), visualized under UV light, and compared to a Quick-Load® Purple 1 kb DNA ladder (#N0552S, New England Biolabs, Germany). Target bands were purified using the Wizard® SV Gel and PCR Clean-Up System (#A9281, Promega, USA) according to the manufacturer’s instructions and subjected to sequencing.

### Determination of cell number and cell size

A total of 1 × 10^5^ WT or KO cells were seeded in 35-mm tissue culture dishes (Sarstedt, Germany). After 24 and 48 h of culture, cells, which grew adherent to the plate, were trypsinized using 0.05% trypsin–EDTA (Gibco, UK) and counted using a Neubauer chamber. The mean cell diameter of trypsinized WT and KO cells was measured using the automated cell counting device CASY System (Omni Life Science, Germany). Measurements were performed as previously described [20].

### Cell cycle analysis and nuclear size assessment

A total of 1 × 10^6^ WT or KO cells were harvested using 0.05% Trypsin–EDTA (Gibco, UK), washed twice with 5 mL cold PBS (Gibco, UK), and centrifuged at 500 *g* for 5 min at 4 °C. The pellet was resuspended in 500 μL PBS to obtain a single-cell suspension, which was slowly added to 4.5 mL ice-cold 70% ethanol (ITW Reagents, Italy) while gently vortexing. Cells were fixed overnight at 4 °C. The next day, cells were centrifuged at 1,000 *g* for 5 min, washed with 5 mL PBS, incubated at RT for 15 min, and centrifuged again at 1,000 *g* for 5 min. The final pellet was resuspended in 300 μL DAPI solution (1 µg/mL in PBS, 0.1% Triton X-100; Sigma-Aldrich, Merck, Germany), and incubated for 30 min at RT, protected from light. Samples were transferred to 1.5 mL tubes for cytometric acquisition using Amnis® ImageStream®X Mk II Imaging Flow Cytometer (Cytek Biosciences, USA) at the Core Facility Flow Cytometry, Biomedical Center, LMU Munich. Data from 2 × 10 cells, with three replicates per cell line, were acquired to assess cell cycle states and nuclear size. Nuclear sizes measured by the flow cytometer are reported in arbitrary units, based on the number of pixels in the DAPI-positive area (and can only be converted to absolute dimensions after calibration). For illustration, nuclear sizes are shown as values relative to WT cells.

### Isolation of RNA, complementary DNA synthesis and quantitative PCR (qPCR)

Total RNA of KGN cells was isolated using the RNeasy Plus Micro Kit (QIAGEN, Germany) according to the manufacturer’s instructions. RNA concentration was determined using the NanoDrop™ 2000/2000c spectrophotometer (Thermo Fisher Scientific, USA). RNA was reverse transcribed using SuperScriptTM II (Invitrogen, Thermo Fisher Scientific, USA). qPCR runs were conducted with the Luna® Universal qPCR Master Mix (New England Biolabs, Germany) on the LightCycler 96® System (Roche Diagnostics, Germany) with 4 ng cDNA. In order to study *FLNA* mRNA expression level in GCT via qPCR, RNA was isolated from four patient samples, as described previously [15]. Primers listed in Suppl. Table 1 were designed using the primer3 tool (http://primer3.wi.mit.edu). Samples were run in duplicate and Cq values evaluated using the ΔΔCq calculation approach [21]. The *L19* gene expression served as an internal control. The specificity of the PCR products was confirmed by sequencing.

### Protein isolation and Western blotting

Cells were lysed with radioimmunoprecipitation assay (RIPA) buffer supplemented with protease and phosphatase inhibitors (Thermo Fisher Scientific, USA). Whole-cell lysates were sonicated and centrifugated at 25,000 *g* at 4 °C for 15 min. Supernatants were collected and the total protein concentration was determined by Lowry assay. Protein samples (10 µg/lane) were separated using 8%, 12% or 4–15% gradient SDS-PAGE and transferred onto nitrocellulose membrane (Amersham™ Protran® 0.45 µm NC; Cytiva, USA). Primary antibodies (FLNA, 1:10,000, #67133-1-Ig, Proteintech, Germany; actin beta (ACTB), 1:5,000, #A5441, Merck, Germany) were incubated overnight at 4 °C. ACTB served as loading control. Protein detection was achieved by incubating the membrane with an adequate IRDye® secondary antibody (1:10,000; 680RD donkey anti-mouse; #92668072, Li-COR Biosciences, USA) for 1 h at RT, followed by detection using Odyssey® CLx (LI-COR Biosciences, USA). Densitometric quantification of the Western blots was performed using software Fiji (version 2.3.0) [22]. The signal of the target protein was normalized to the loading control for quantification.

### Immunocytochemistry

WT or KO cells were seeded onto round 12-mm glass coverslips (2 × 10^4^ cells/coverslip). After attachment, cells were washed with PBS and fixed with buffered 4% formaldehyde solution, pH 6.9 (Merck, Germany) for 10 min. Following fixation, the cells were washed three times with PBS and then incubated for 30 min at RT in blocking buffer consisting of PBS supplemented with 5% normal goat serum and 0.1% Triton X-100 (both from Sigma-Aldrich, Merck, Germany). After blocking, the cells were incubated with primary antibodies diluted in blocking buffer (FLNA: 1:1000, #67133-1-Ig, Proteintech, Germany) overnight at 4 °C, followed by the incubation with secondary goat anti-mouse Alexa Fluor 647 (1:1000, #21236, Thermo Fisher Scientific, USA) diluted in blocking buffer for 1 h at RT. Phalloidin-Atto 550 (1:500 diluted in PBS, #19083, Merck, Germany) and DAPI (1 µg/mL, diluted in H_2_O, Sigma-Aldrich, Merck, Germany) were used to visualize actin filaments and DNA, respectively. After immunostaining, samples were washed in PBS and water and subsequently embedded using Dako mounting medium (Agilent Technologies, USA). Images were acquired using a Zeiss Z1 Axio Observer microscope equipped with a 63×/1.4 NA PlanApo oil immersion objective (Carl Zeiss Microscopy, Germany) and ZEN acquisition software (ZEN blue 2.6).

### Wound-healing scratch assay and determination of migration velocity

A total of 2 × 10^4^ WT or 1.5 × 10^4^ KO cells were seeded into each well of a culture-insert 2-well in a 35 mm μ-Dish (ibiTreat, ibidi, Germany) for the wound-healing scratch assay. For random movement experiments 9 × 10^4^ WT or 5 × 10^4^ KO cells were seeded into a 35 mm μ-Dish. To ensure equivalent confluence of both cell types at the start of this experiment, different amounts of cells were seeded. After cell attachment, the inserts were removed for the wound-healing scratch assay, whereas for the random movement experiments no inserts were used. In both cases, the dishes were washed with 1 mL PBS, and then 2 mL DMEM/F12 medium containing 10% FCS and 0.1 µg/mL Normocin was added.

Imaging was performed in a Stage Top Incubator (ibidi, Germany) at 37 °C, 5% CO_2_ and 95% humidity, integrated into an inverted microscope (Axiovert 135 TV, Carl Zeiss Microscopy, Germany) equipped with a ProgRes® MF cool camera (JENOPTIK, Germany).

In the wound-healing scratch assay, cell migration was recorded at 20-min intervals over 72 h using the software ProgRes® CapturePro (version 2.9.0.1). The percentage of confluence in the wound area was quantified every 10 h over a period of 70 h using the free accessible ImageJ/Fiji plugin PHANTAST (https://github.com/nicjac/PHANTAST-FIJI/wiki/PHANTAST-FIJI-plugin-tutorial). Recordings for random motility analysis were carried out at 20-min intervals over 48 h. Cell migration paths were tracked using the ImageJ/Fiji plugin Manual Tracking (developed by Fabrice Cordelières, Institut Curie, available from: https://imagej.net/ij/plugins/track/track.html), and migration velocity directionality were estimated using the plugin Chemotaxis Tool (freely available on the homepage of ibidi GmbH: https://ibidi.com/manual-image-analysis/171-chemotaxis-and-migration-tool.html).

### Shear stress experiments and image analysis

A total of 1.8 × 10^5^ WT or 1.2 × 10^5^ KO cells were seeded in a μ-Slide I^0.4^ Luer (ibiTreat, ibidi, Germany). Again, in order to ensure comparable confluence of both cell types at the beginning of the experiment, different amounts of cells were seeded. After overnight incubation, attached cells were exposed to a constant laminar flow using the ibidi pump system, applying a shear force of 25 dyn/cm^2^ for 24 h, which was determined based on preliminary experiments. Thereon, the cells were either harvested for qPCR analysis or subjected to fluorescence staining with phalloidin-Atto 550 (1:500 diluted in PBS, #19083, Merck, Germany) and DAPI (1 µg/mL, diluted in H_2_O, Sigma-Aldrich, Merck, Germany).

Analysis of actin filament orientation was performed using the freely accessible ImageJ/Fiji plugin OrientationJ (https://bigwww.epfl.ch/demo/orientation/#soft). This tool evaluates orientation at each pixel based on the structure tensor. The function Color Survey was used to generate a color-coded orientation map of actin filaments, while the function Distribution produced a histogram representing the distribution of actin filament orientations.

### Mass spectrometric analysis

Cells were lysed in 8 M urea/50 mM NH_4_HCO_3_ and sonicated (Sonopuls cup resonator, Bandelin, Germany) for 15 min. For complete cell lysis, QIAshredder homogenizers (QIAGEN, Germany) were used. Protein concentrations were determined using the Pierce 660 nm protein assay kit (Thermo Fisher Scientific, USA). Prior to digestion, cysteine residues were reduced with 1,4-dithiothreitol (5 mM for 30 min at 56 °C) and carbamidomethylated for 30 min at RT at an iodoacetamide concentration of 15 mM. Quenching was performed at 10 mM 1,4-dithiothreitol for 15 min. For protein digestion the following steps were performed: (a) 4 h digestion at 37 °C with lysyl-endopeptidase C (1:100 enzyme/protein ratio, Fujifilm Wako, Germany), (b) dilution with 50 mM NH_4_HCO_3_ to 1 M urea and (c) overnight digestion with modified porcine trypsin at 37 °C (1:50 enzyme/protein ratio, Promega, USA). Liquid chromatography–tandem mass spectrometry (LC–MS/MS) was done with an Ultimate 3000 RSLC chromatography system and a Q Exactive HF-X mass spectrometer (Thermo Fisher Scientific, USA). Samples were injected onto a PepMap 100 C18 trap column (100 µm × 2 cm, 5 µM particles, Thermo Fisher Scientific, USA) and separated on an analytical column (PepMap RSLC C18, 75 µm × 50 cm, 2 µm particles; Thermo Fisher Scientific, USA) at a flow rate of 250 nL/min. Solvent A was 0.1% formic acid in water and solvent B 0.1% formic acid in acetonitrile. The following chromatography method was used: (i) 80 min gradient from 5–20% of solvent B, (ii) 9 min gradient 20–40% solvent B, (iii) 9 min isocratic flow at 85% solvent B and (iv) a 10 min re-equilibration step with 3% solvent B. The Q-Exactive HF-X instrument was operated in the DIA mode and acquired 50 × 12 m/z-wide windows in the range of 400–1000 m/z using a staggered pattern. Raw files were processed using DIA-NN (v1.8). For protein identification and quantification, predicted spectral libraries generated in DIA-NN from the human subset of the UniProt database were used.

### Gene ontology analysis

Differentially abundant proteins identified by mass spectrometry were subjected to Gene Ontology (GO) enrichment analysis using the Database for Annotation, Visualization and Integrated Discovery (DAVID) [23, 24]. Enrichment was assessed within the GOTERM_BP_DIRECT category to identify overrepresented biological processes using the following thresholds: Count ≥ 20 and EASE score ≤ 0.05.

### Steroid measurement

WT and KO cells were seeded and cultured in 60-mm tissue culture dishes (Sarstedt, Germany). When cells reached a confluence of 80%, the medium was changed, and supernatants were collected after another 24-h culturing. Samples were analyzed using LC–MS/MS, as described elsewhere [25]. The corresponding amount of protein of cell mass was determined and used for normalization as described recently [26].

### Statistics

Statistical analysis was performed using GraphPad Prism 9.0 (GraphPad Software, San Diego, CA, USA). Data sets were checked for outliers (ROUT method, Q = 1%) and Gaussian distribution (Shapiro–Wilk normality test, α = 0.05) before further analysis. For comparisons within one cell type, unpaired t-tests (two-tailed) or Mann–Whitney tests were used, when Gaussian distribution could not be assumed. For comparisons between WT and KO cells, a one-way ANOVA or two-way ANOVA with multiple comparisons (WT vs. each KO) was used. When Gaussian distribution could not be assumed, a non-parametric Kruskal–Wallis test was applied. For all analyses, statistical significance was set at *p* < 0.05 (*p ≤ 0.05, ** *p* ≤ 0.01, *** *p* ≤ 0.001, **** *p* ≤ 0.0001).

## Results

### FLNA expression in granulosa cell tumors (GCTs)

**Fig. 1:**
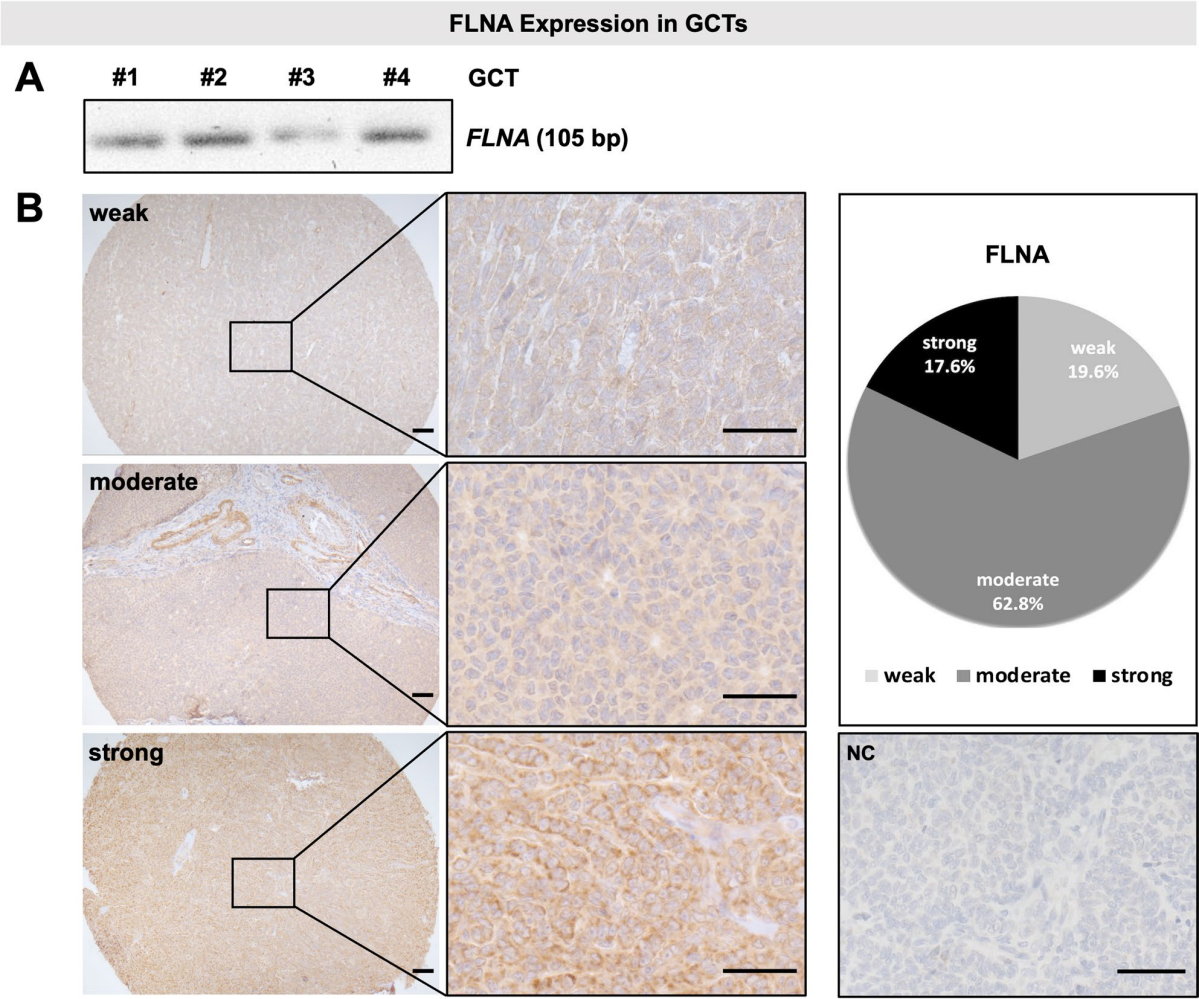
FLNA expression in granulosa cell tumors (GCTs). **A** qPCR revealed *FLNA* mRNA (single band 105 bp) in GCT samples. The original gel image is shown in Suppl. Fig. S1. **B** Immunohistochemical staining and evaluation of FLNA expression in tissue microarrays of GCT samples. The samples exhibited expression of FLNA protein in their cytoplasm. Three representative images of different staining intensities designated as weak, moderate and strong, are shown. NC: negative control (IgG); scale bars: 50 µm

To evaluate *FLNA* gene expression in GCTs, we first conducted a qPCR screening using samples from four individual patients, all of which showed *FLNA* expression (Fig. 1A, Suppl. Fig. S1A). Immunohistochemical staining of tissue microarrays (TMAs) of 51 GCT samples revealed exclusively cytoplasmatic localization of FLNA protein. Therefore, a simplified Remmele and Stegner score was applied for staining patterns and designated as weak, moderate and strong. Evaluation of tumor staining by two researchers revealed 32 samples (62.8%) with moderate FLNA expression, 10 tumors, which exhibited weak FLNA staining (19.6%), while in 9 samples (17.6%) strong immunoreactive signals were observed. As a negative control, non-immune mouse IgG was used instead of the primary antibody. Representative immunohistochemical images and quantitative analyses are shown in Fig. 1B. Available clinical parameters, including the Ki67 proliferation index, tumor status (primary versus recurrent; available for 37 tumors), and tumor stage (available for 34 tumors) are summarized in Suppl. Fig. S1B. No clear correlation between FLNA immunostaining and these parameters was observed, which may be due to the restricted sample size in certain subgroups and the availability of clinical annotations.

### Verification of FLNA-deficient KGN cells

**Fig. 2:**
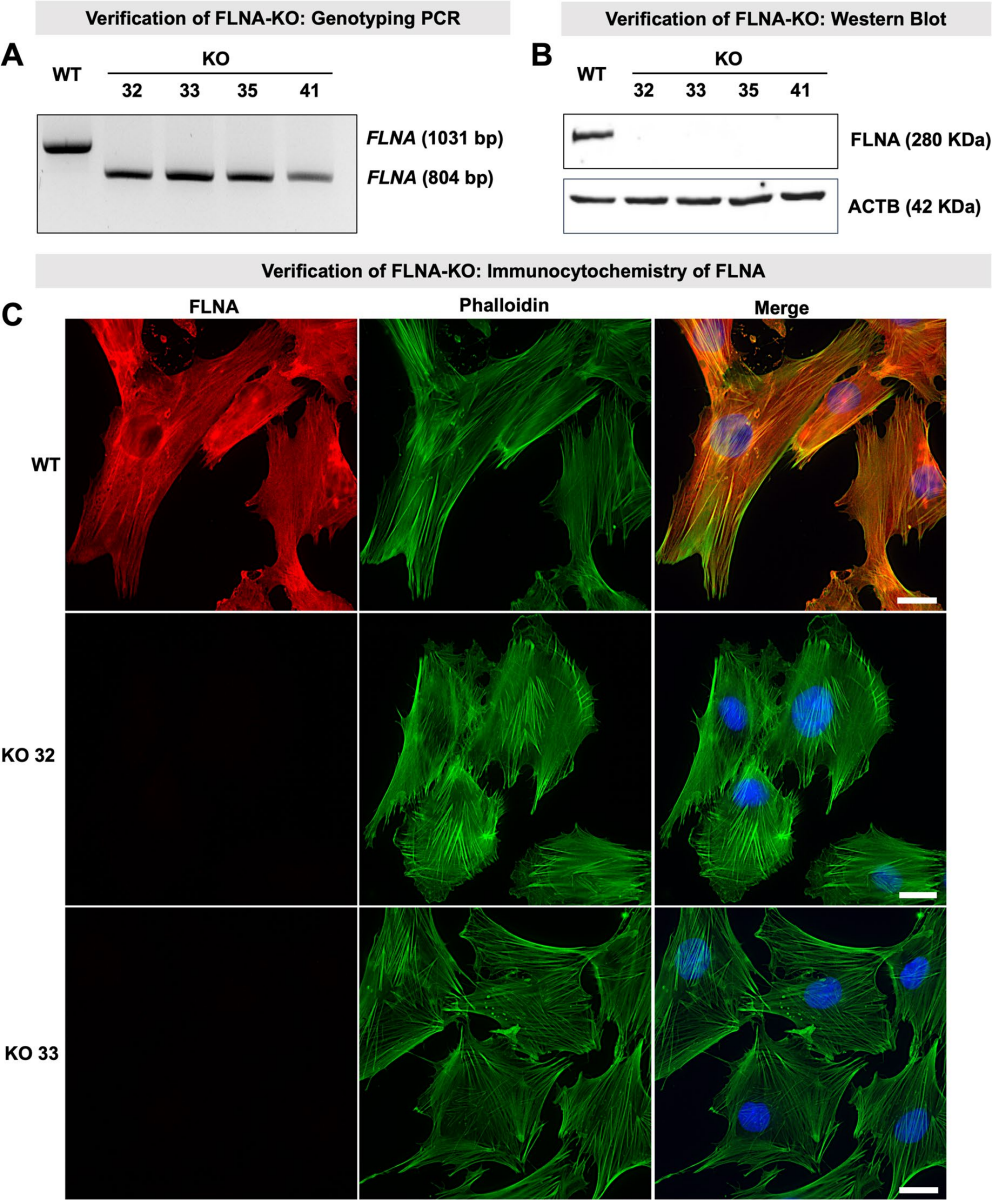
Verification of FLNA-knockout (KO) KGN cells. **A** Agarose gel electrophoresis of *FLNA* genotyping PCR using a primer pair designed to amplify a 1031 bp fragment. Wildtype (WT) cells showed a full-length PCR product, while all four FLNA-KO clones (KO 32, KO 33, KO 35 and KO 41) exhibited a truncated product, confirming successful gene editing. Original gel image is shown in Suppl. Fig. S2A. **B** FLNA protein was detected in WT cells but was absent in all FLNA-KO clones. ACTB was used as a loading control. Original blot images are shown in Suppl. Fig. S2B. **C** Cells were stained with phalloidin-Atto 550 (green) to label actin, immunolabeled with FLNA-specific primary antibodies and Alexa-647-labeled secondary antibodies (red) to detect FLNA, and counterstained with DAPI (blue, shown in merge) to visualize nuclei. In WT cells, FLNA exhibited a filamentous staining pattern that largely colocalized with actin filaments. In contrast, FLNA staining was absent in FLNA-KO cells (only KO 32 and KO 33 are shown), confirming the loss of FLNA expression. Scale bars: 20 µm

Four FLNA-knockout (KO) clones (named KO 32, 33, 35 and 41) were generated using the CRISPR/Cas9 technology. Successful gene editing was confirmed by PCR-based genotyping with primers flanking the targeted region. WT cells yielded the expected full-length PCR product of 1031 bp, whereas all four KO clones showed a shorter, truncated fragment (Fig. 2A and Suppl. Fig. S2A). DNA sequencing verified successful genomic editing (Suppl. Fig. S2C).

Western blot analysis confirmed robust FLNA expression in WT cells, while FLNA protein was undetectable in all KO clones (Fig. 2B and Suppl. Fig. S2B). Immunocytochemistry revealed a filamentous FLNA staining pattern. It strongly colocalized with actin stress fibers but also showed additional cytoplasmic distribution. No FLNA staining was detected in KO cells (Fig. 2C). Since the analyses revealed very high similarity of all four clones, further experiments were performed with at least two clones, namely, KO 32 and KO 33, unless indicated otherwise.

Of note, FLNB levels remained unaltered in KO cells, as confirmed by mass spectrometric (MS) analyses and Western blotting (Suppl. Fig. S2D–E).

### FLNA deficiency leads to significant changes in the proteom of KGN cells

**Fig. 3:**
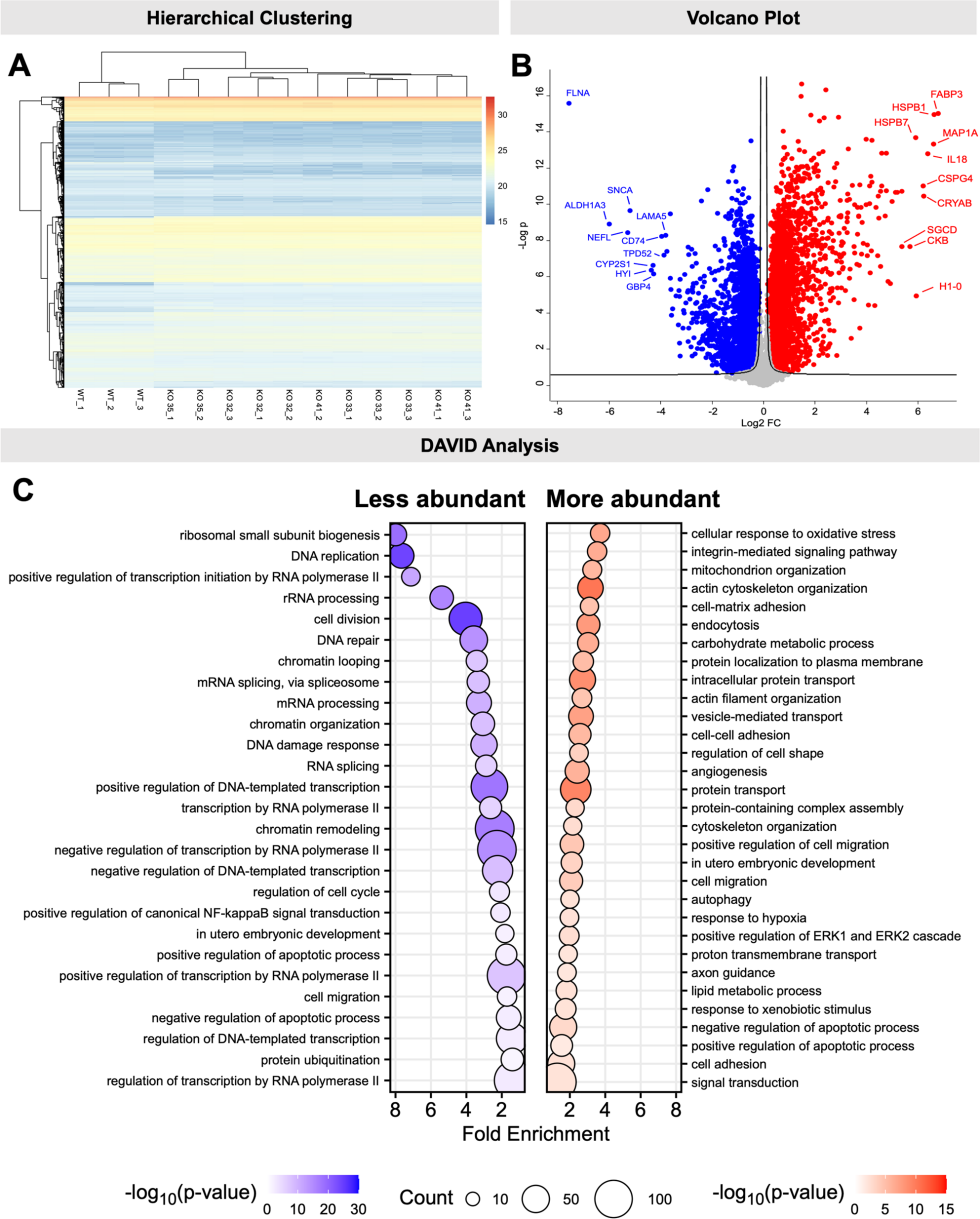
Proteomic analysis of FLNA-knockout (KO) KGN cells. **A** The Heatmap displays hierarchical clustering of differentially abundant proteins across samples. The color scale indicates protein abundance, with blue representing lower abundance and red representing higher abundance. Clustering reveals distinct patterns of protein expression between wildtype (WT) cells and FLNA-KO cells (KO 32, KO 33, KO 35 and KO 41). **B** The volcano plot displays differentially abundant proteins, with each point representing a protein. A total of 6,413 proteins were identified, with 979 showing significant lower abundance and 1211 showing higher abundance (q-value < 0.05, log2 fold change ≤ − 0.6 or ≥ 0.6). Proteins which were differentially abundant are highlighted in blue (downregulated) and red (upregulated). The outlier from KO 35 was excluded from the analysis. Black curves represent the permutation-based FDR significance cutoff. The ten proteins with the largest increases (red) and decreases (blue) in abundance are marked. **C** Overrepresentation analysis using DAVID and the “Biological Process” subset of the GO database of proteins with decreased (left panel) or increased abundance (right panel). The size of each bubble represents the number of genes associated with each term, and the color intensity indicates the significance of enrichment

We performed MS analyses to identify proteins differentially expressed in KO compared to WT cells. Three technical replicates of WT and each of the four KO clones were analyzed. A total of 6,413 proteins were quantified (Suppl. Table 2). Unsupervised hierarchical clustering clearly showed a separation in two distinct groups, namely KO and WT cells (Fig. 3A). Furthermore, the Volcano plot analysis revealed prominent alterations in the proteome profile of KO cells in comparison to WT cells. In total, 979 proteins showed significantly decreased abundance and 1,211 proteins exhibited significantly increased abundance (FDR < 0.05, |log2 fold change|≤ 0.6) in KO cells compared to WT cells (Fig. 2B, Suppl. Table 2). Gene Ontology (GO) enrichment analysis (focusing on biological processes) of differentially abundant proteins revealed that proteins significantly overrepresented in abundance were related to adhesion (related GO terms: cell–matrix adhesion and cell–cell adhesion), actin cytoskeleton organization, regulation of cell shape, and lipid metabolism (Fig. 3C, right panel, Table 1 and Suppl. Table 3). In contrast, downregulated proteins were significantly enriched in terms including DNA replication, cell division and regulation of cell cycle. Moreover, proteins of pathways connected to ribosomal small subunit biogenesis, chromatin remodeling, RNA processing (related GO terms: rRNA processing, mRNA processing) were also decreased in abundance (Fig. 3C, left panel, Table 2 and Suppl. Table 3). GO analysis of cellular components further identified selective upregulation of proteins linked to focal adhesion and cell-substrate junctions, while proteins with lower abundance were mainly related to the nucleus and chromatin (Suppl. Fig. S3A–B).

**Table 1:** Summary of proteins with higher abundance in FLNA-knockout KGN cells

Enriched term	Fold Enrichment	Annotated proteins
Actin cytoskeleton organization	3.16	SPTAN1, ACTN1, SPECC1L, PACSIN2, PDE4DIP, CORO1B, MYH9, WASL, ADD1, EZR, BCR, CDK5, NF2, ANXA1, KRAS, RND3, ARHGEF17, TRIP10, MRTFA, CAPZA2, NHERF1, KANK1, SIPA1L1, CORO2B, SSH1, MICALL2, DMD, SPTBN1, PDLIM2, PDLIM7, INPP5K, ACTN4, MINK1, PALLD, FMN2, IQSEC1, EHBP1, PARVB, OPHN1, ITPKA, PDLIM3, PAK1, INF2
Cell–matrix adhesion	3.11	ITGB8, ITGA5, ITGB1, ZYX, COL3A1, ADAM15, ITGAV, PPFIA1, EPDR1, ITGB5, SGCE, CTNNB1, ITGA4, FERMT2, CDK5, FERMT1, ANXA2, ITGA7, CD63, ITGA3
Cell–cell adhesion	2.58	TMOD3, THY1, ITGAV, ITGB5, PLPP3, LIMS2, DSP, CD58, CTNNA1, LIMS4, ANXA1, RAB10, ITGA7, ANXA2, ITGA5, ITGB8, ITGB1, FAT1, CDH2, DLG1, CTNNB1, ITGA4, STXBP6, CDC42EP1, SCRIB, LGALS1, S100A11, PKP2, LIMS3, TMEM47, ITGA3
Regulation of cell shape	2.52	PLXNB2, FMNL1, PALMD, DLG1, GNA12, MYH9, CDC42EP4, MYH14, EZR, CDC42EP1, PLXNA2, FERMT2, TPM1, NF2, ANXA1, FYN, GNA13, MSN, ITGA7, TPM3, NHERF1
Cytoskeleton organization	2.17	MICAL2, FMNL1, THY1, FITM2, SH3D19, ANK3, SORBS2, MICAL3, PACSIN2, ARHGAP21, DMD, ANK2, SH3BGRL3, ADD1, ABLIM1, PALLD, ADD3, TPM1, MSN, TPM3
Lipid metabolic process	1.81	HMGCL, FADS3, GSTM4, CRKL, FABP5, MGLL, PAFAH2, EPHX1, B4GALT1, CYP51A1, SDSL, ALDH3B1, HSPG2, APOL2, PTPRN2, ACOX1, LRP10, GDE1, MGST3, G6PD, ABHD11, ARSA, PLCD3, PITPNB, ACOT13, LRP1, BTN2A1
Cell adhesion	1.51	COL1A1, ITGB5, SPECC1L, CD47, CTNNA1, FERMT2, CD9, PODXL, AFDN, B4GALT1, RND3, ITGA7, DPP4, ITGA5, COL6A1, DDR2, ADGRE5, PPFIBP1, CDH2, MFGE8, CD99L2, ATP1B1, FAP, ARHGAP5, THY1, ITGAV, CD151, MXRA8, CERCAM, ITGB8, ITGB1, EPHA4, PCDH1, ALCAM, FAT1, EPHB4, SSPN, GPNMB, CTNNB1, ITGA4, PBXIP1, SUSD5, PARVB, RHOB, TNC, MCAM, COL8A1, FERMT1, ITGA3

**Table 2:** Summary of proteins with lower in abundance in FLNA-knockout KGN cells

Enriched term	Fold Enrichment	Annotated proteins
Ribosomal small subunit biogenesis	7.99	EMG1, NAT10, UTP6, TBL3, HEATR1, AATF, MPHOSPH10, RRP7A, UTP15, WDR43, WDR36, AK5, BMS1, UTP4, LTV1, DIMT1, PWP2, RIOK2, DNTTIP2, EXOSC10, DHX37, RCL1, IMP4, UTP18, IMP3, UTP20, WDR75, NOB1, WDR3
DNA replication	7.66	MCM6, RPA1, DUT, RPA2, NASP, POLD1, MCM5, MCM7, MCM4, FAM111A, LIG1, GINS1, RBBP7, PCLAF, MCM2, SAMHD1, SDE2, GRWD1, RFC5, FEN1, RPA3, RBBP4, CHTF18, RNASEH2A, RFC4, DHX9, SIN3A, POLA2, SSRP1, KIN, CDK2, CDK1, POLD3, POLA1, DTL, POLD2, CHAF1A, CHAF1B, CHEK1, SUPT16H, POLE3
rRNA processing	5.39	BYSL, EMG1, NVL, TBL3, ESF1, DDX47, EXOSC9, HEATR1, MRTO4, MPHOSPH10, RRP7A, UTP15, EXOSC6, DCAF13, MTREX, WDR36, AK5, NOC4L, EXOSC7, FRG1, PIN4, DIS3, EXOSC2, NOLC1, EXOSC10, EXOSC3, GEMIN4, IMP4, UTP18, IMP3, UTP20, UTP14A, WDR75, EXOSC4, NOB1
Cell division	4.04	ZNF207, LATS1, NUDC, PTTG1, CKAP5, CDK4, CKS2, MCM5, CDK7, CCNB1, PARD3B, RCC2, SPOUT1, BUB1B, MCMBP, CKS1B, NCAPG2, EPB41, CCNT1, NCAPD2, STAG2, TPX2, HNRNPU, NEK6, CDCA8, CDC20, BUB3, NUF2, SSNA1, NRDE2, UBE2S, PMF1, NCAPD3, SKA1, KIFC1, PDS5A, WAPL, ERCC6L, MAPRE2, MAD2L1, NCAPG, UBE2C, POGZ, KIF11, VRK1, LIG1, KIF2C, EML4, TXNL4A, TACC3, NCAPH, AURKA, RB1, SPDL1, SDE2, SMC2, CDK19, ZNF830, ZWINT, NSL1, PRC1, PLK1, MPLKIP, BCAR1, DSN1, MTCL1, SMC4, NR3C1, CDK2, SPART, CDK1, CDCA3, NDC80, KIF14, SMC1A
mRNA processing	3.29	HNRNPF, TSEN34, PAPOLA, PPWD1, CELF1, NSRP1, CMTR1, SMNDC1, PUS7L, PNN, RBMXL1, JMJD6, GRSF1, DDX47, SAP18, CPSF3, SUPT6H, CCAR2, SYMPK, SFPQ, CNOT6L, DHX15, PDE12, RPUSD2, ZNF830, METTL16, DCPS, CRIPT, THOC3, KHDRBS1, NRDE2, CHD8, RBM38, PTBP2, KIN, HNRNPA0, ECD, IVNS1ABP, NONO, MBNL2
chromatin remodeling	2.40	BRD9, MCM6, MIER1, LATS1, RPS6KA1, MCM5, MCM7, INO80C, MCM4, BUB1B, MCM3, NEK6, SLFN11, TTK, TNIK, WRNIP1, CSK, EGFR, MYO3B, NSD2, PTPN14, NCOA3, GTF3C4, PIKFYVE, DDX47, AQR, VRK1, CREBBP, MTA3, SMARCD1, MTREX, EYA3, SFPQ, RB1, AURKA, DHX15, ERCC2, HDAC1, INO80B, DHX37, PSIP1, DDX20, SMARCA4, PHF10, BMI1, ROR2, TAF6L, PRKCD, DPF2, CDK1, KDM5C, UBASH3B, DDX41, YY1, ATM, DDX50, LYN, JMJD6, KDM2A, DHX36, ERCC6, CHD3, SMARCD2, SMARCE1, ACTR6, HDAC2, PBK, KSR1, CFDP1, BAZ1A, SETD1A, PAK2, SMARCC1, CHEK1, MTA1, ERCC6L, USP36, USP22, BAZ2A, KDM4A, RSBN1, RSF1, DDX5, CBX3, IGF1R, SUDS3, RBBP7, RYBP, CHD1, DCLK1, EPHB2, YEATS2, RIOK2, GATAD2A, RBBP4, NIPBL, AXL, CHD8, DHX9, DCAF1, ARID2, CDK2, ARID1A, SS18, EPHA2, DHX8, POLE3
regulation of cell cycle	2.13	ZNF703, YY1, ATM, CDK4, CDK7, INO80C, TXLNG, PRR11, PCLAF, PRCC, RB1, JUN, CDK19, YEATS2, PLK1, KHDRBS1, INO80B, JUND, SGF29, MRGBP, RBM38, TFDP1, BRD8, DTL, MORF4L2

### Disruption of the *FLNA* gene results in changes of cell morphology, cell proliferation, cell cycle progression and steroid production of KGN cells

**Fig. 4:**
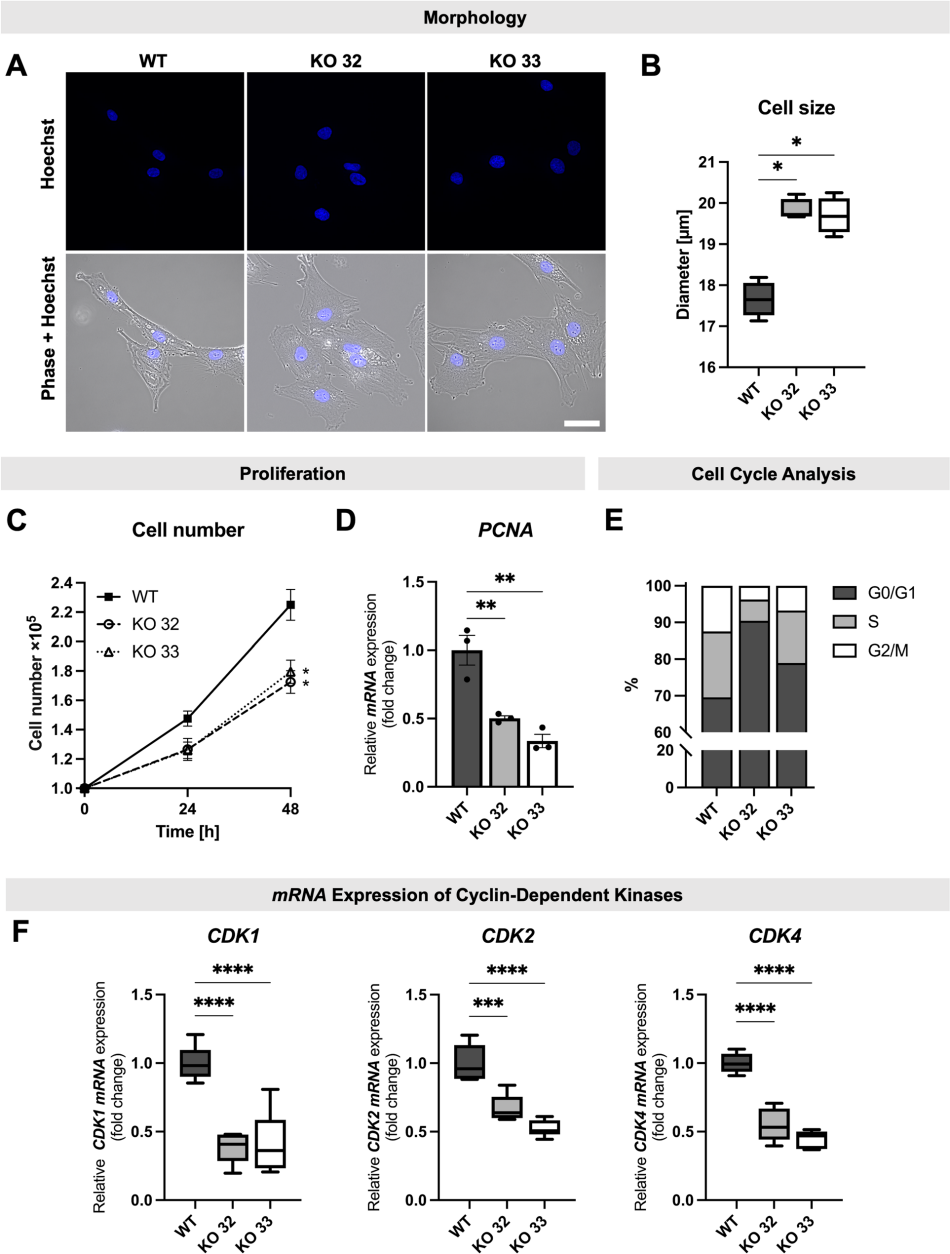
FLNA-knockout (KO) affects morphology, and impairs proliferation and cell cycle progression of KGN cells. **A** Cell morphology: Fluorescence images of nuclei (upper panel) and merged phase-contrast images (lower panel) of wildtype (WT) and FLNA-KO cells (KO 32 and KO 33) reveal a change to a flattened morphology in FLNA-KO cells. Scale bar: 50 µm. **B** Cell size: FLNA-KO cells exhibited significantly larger cell diameters compared to WT cells. Data are presented as min-to-max box-and-whisker plots, with the box representing the interquartile range (25%–75%) and the median inside; n = 4–8 (n: number of measurements used to determine the mean diameter of trypsinized WT or FLNA-KO cells, Kruskal–Wallis test (* p < 0.05). **C** Cell number: FLNA-KO cells demonstrated a significantly lower proliferation rate compared to WT cells. Cell counts were measured at 24 h and 48 h post-seeding (1 × 10^5^ cells). After 48 h, WT cells exhibited a significantly higher cell count compared to FLNA-KO cells; mean ± SEM (n = 4), two-way ANOVA (* *p* < 0.05). **D** *PCNA* expression: FLNA-KO cells exhibited significantly reduced mRNA expression levels of the proliferation marker *PCNA*, as determined by quantitative PCR (qPCR) analysis; mean ± SEM (n = 3), one-way ANOVA (** *p* < 0.01). **E** Cell cycle Analysis: Flow cytometry analysis revealed an increased G0/G1 subpopulation and a decreased G2/M subpopulation in FLNA-KO cells compared to WT cells. (n = 3, 2 × 10^4^ cells per replicate and cell type). **F** mRNA expression of cyclin-dependent kinases: FLNA-KO cells exhibited significantly lower mRNA levels of *CDK1*, *CDK2* and *CDK4*, as determined by qPCR analysis; min-to-max box-and-whisker plots, with the box representing the interquartile range (25%–75%) and the median inside (n = 6), one-way ANOVA (**** p < 0.0001)

FLNA-deficient cells exhibited a markedly altered phenotype. Compared to the spindle-shaped WT cells, KO cells adopted a more flattened cell shape (Fig. 4A, see also Fig. 2C), and showed a significant increase in cell diameter (Fig. 4B). Further evaluation of nuclear size revealed that KO cells had significantly smaller nuclei (Suppl. Fig. S4A). Moreover, FLNA deficiency led to a decrease in proliferation rate, which is indicated by a reduction in cell number by 23.3% (KO 32) and 20.2% (KO 33) over a 48-h culture period (Fig. 4C). This finding was corroborated at the mRNA level of the proliferating cell nuclear antigen (*PCNA*), a molecular marker of cell proliferation, which was significantly reduced in KO cells with reduction to 50.2% in KO 32 and 43.6% in KO 33 relative to WT expression levels (Fig. 4D). The reduction of PCNA mRNA in each individual KO clone was also reflected by quantitative MS data (Suppl. Fig. S3C). Cell cycle analyses further revealed an increase in the G0/G1 subpopulation in KO cells (WT: 69.6% vs KO 32: 90.4% vs KO 33: 78.9%) and a decrease in G2/M subpopulation (WT: 12.5% vs KO 32: 3.8% vs KO 33: 6.8%) (Fig. 4E). This shift in cell cycle progression was accompanied by significantly lower mRNA expression levels of the cyclin-dependent kinases *CDK1*, *CDK2*, and *CDK4*, in both KO clones compared to WT cells (Fig. 4F).

To explore changes in the steroidogenic potential of KGN cells, cell culture supernatant was harvested from WT and KO cells after 24 h of culture under standard conditions and subjected to LC–MS/MS analysis. Pregnenolone levels did not differ significantly between WT and KO cells. Progesterone was significantly increased in KO 32 (threefold compared to WT), while KO 33 showed a similar, non-significant trend (1.9-fold) (Suppl. Fig. S4B).

### Disruption of the *FLNA* gene impairs motility of KGN cells

**Fig. 5:**
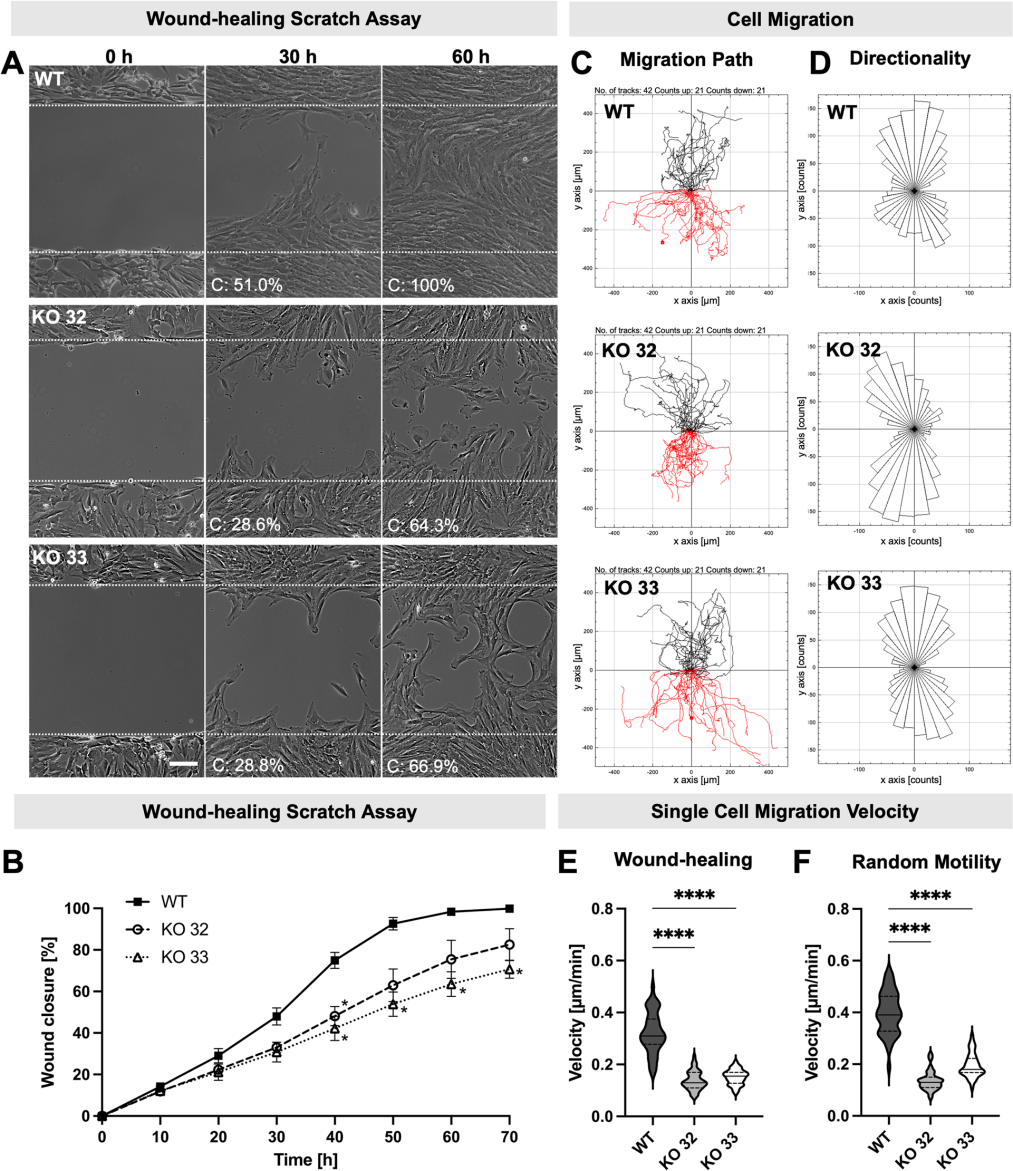
FLNA-knockout (KO) impairs KGN cell migration and reduce migration velocity. **A** Wound-healing scratch assay: Representative phase-contrast images of the wound-healing scratch assay performed with wildtype (WT) and FLNA-KO (KO 32 and KO 33) cells at 0-, 30-, and 60 h post-scratching. The percentage of confluence (C) within the wound area is indicated. Scale bar: 100 µm. Time-lapse recordings of the assay are provided in Supplementary Videos 1–3. **B** Wound-healing over time: The percentage of wound closure (confluence in the scratch area) was quantified for WT and FLNA-KO cells over a 0–70 h period; mean ± SEM (n = 3), two-way ANOVA (* *p* < 0.05). **C** Migration path: Spatial tracking of WT and FLNA-KO cells during the wound-healing scratch assay. All tracked cells are assumed to start from the coordinate origin. Red indicates cells migrating downward, while black represents cells migrating upward. **D** Directionality: Rose plots of the migration trajectories of WT and FLNA-KO cells during the wound-healing scratch assay. The plots illustrate the distribution of movement directions, with both WT and FLNA-KO cells showing a strong vertical orientation. **E** Directed migration velocity: The migration velocity during the wound-healing scratch assay of WT and FLNA-KO cells was determined; mean ± SEM (n = 42). The solid line represents the median, and the dashed lines indicate the quartiles, one-way ANOVA (**** p < 0.0001). **F** Random motility velocity: The motility velocity of WT and FLNA-KO cells (KO 32 and KO 33) was assessed for random movement experiments; mean ± SEM (n = 42). The solid line represents the median, and the dashed lines indicate the quartiles, a one-way ANOVA (**** *p* < 0.0001)

To study consequences of FLNA disruption on cell migration, a scratch assay was performed. Disruption of the *FLNA* gene led to a significantly decreased motility of KGN cells over a 72-h observation period. In Fig. 5A, representative recordings for WT and KO 32 and KO 33 cells are shown. Quantitative analysis of confluence, using the ImageJ/Fiji PHANTAST plug-in at 30 and 60 h, confirmed that KO cells were markedly less efficient than WT cells at closing the gap (confluence (C): expressed as the percentage of confluence within the wound area. At 30 h: 51% for WT versus 28.05–36.3% for KO; at 60 h: 100% for WT versus 62.1%–68.1% for KO; see also Suppl. Video 1–3). Over a 70-h time course, a sustained delay in wound closure by KO clones was evident (Fig. 5B). Since differences in cell proliferation rates can affect the outcome of confluence-based scratch assays, we performed single-cell tracking to more accurately assess migratory behavior. Trajectory and rose plots illustrate the migration paths and directionality of individual cells (Fig. 5C–D). Notably, FLNA-KO did not affect the directionality of migration, as the distribution of movement remained aligned along the vertical axis for both WT and KO cells. However, quantitative analysis of single-cell migration velocity revealed a significant reduction of the speed of KO cells by 57.2% (KO 32) and 53.1% (KO 33) (Fig. 5E). Random motility analysis also showed that KO cells moved more slowly than WT cells. This finding was determined by tracking individual cell movements over a 48-h period. (Fig. 5F and Suppl. Videos 4–6). Of note, KO cells exhibited a distinct change in their shape at the leading edge (front), forming broad lamellipodia that expand into free (scratched) areas. This structural feature was consistently observed in both KO clones during directed migration and random motility assays (Fig. 5A and Suppl. Videos 2–3, 5–6).

### Disruption of the *FLNA* gene affects cell orientation and expression of FLNA-associated genes in response to mechanical stress

Since FLNA is involved in mechanosensing, we next investigated how FLNA disruption affects the cellular interaction with its environment in response to mechanical cues. Therefore, KGN cells were exposed to either static conditions or high laminar shear stress (25 dyn/cm^2^) for 24 h in a flow chamber, followed by phalloidin-staining to highlight the arrangement of filamentous actin structures.

**Fig. 6:**
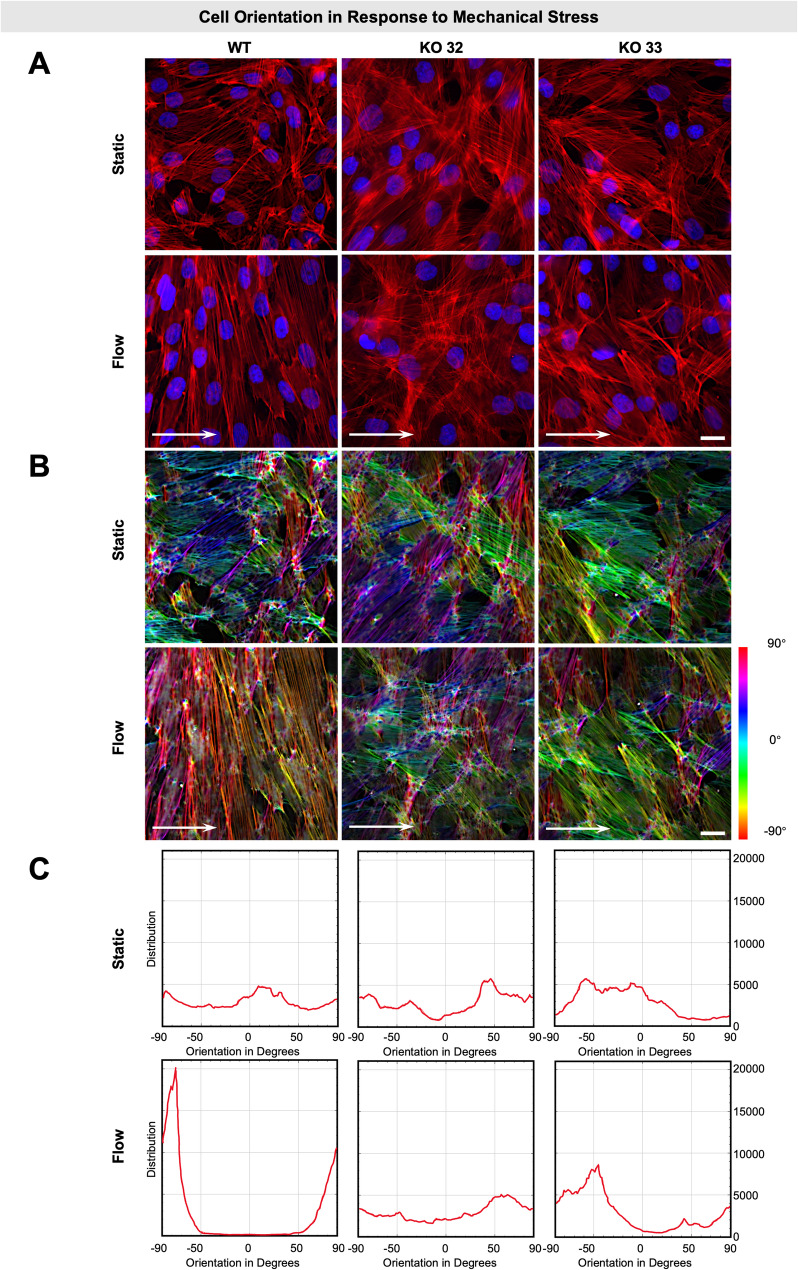
Actin cytoskeletal response to laminar flow shear stress. **A** Actin cytoskeleton reorganization: Wildtype (WT) and FLNA-knockout (KO) cells were subjected to unidirectional laminar shear stress (25 dyn/cm^2^ for 24 h). As a control, cells were cultured under static conditions without flow for 24 h. Cells were stained with phalloidin-Atto 550 (red) to label actin filaments and counterstained with DAPI (blue) to visualize nuclei. WT cells reoriented their actin cytoskeleton and aligned in a vertical direction to the flow after exposure to shear stress, as indicated by the arrows showing the flow direction. In contrast, FLNA-KO cells failed to reorientate, demonstrating a lack of directional alignment in response to shear stress. Scale bars: 20 µm. **B** Color-coded orientation map of actin filaments: Actin filament orientations (− 90° to 90°) were visualized using color-coded maps. A more homogeneous color distribution indicates a dominant alignment direction. **C** Quantitative analysis of actin filament orientation: Distribution of actin filament orientations were quantified for each cell type and condition. Graphs display the frequency of actin filament orientations (y-axis) relative to angular orientation in degrees (− 90° to 90°, x-axis)

Under static conditions, orientation of both WT and KO cells was rather random without specific alignment, as visualized for actin filaments by phalloidin-staining (Fig. 6A, upper panel). In WT cells, exposure to high shear stress for 24 h induced a perpendicular alignment of actin filaments relative to the flow direction. Signs of reorganization and vertical alignment appeared as early as 12 h after flow onset, although full alignment had not yet occurred. In contrast, KO cells failed to reorient under flow in such a way. They rather retained a random, omnidirectional actin filament orientation similar to static conditions (Fig. 6A, lower panel).

For in-depth orientation analysis and quantification of the distribution of phalloidin-stained filaments alignment between both cell types under each experimental condition, we used the software plugin tool OrientationJ (ImageJ/Fiji). The color-coded orientation images (Fig. 6B) and histograms of corresponding distribution of orientation (Fig. 6C) confirmed the markedly impaired ability of KO cells to align actin filaments under flow.

Since FLNA is a central hub for multiple interacting partners, we further asked whether its presence or absence affects the expression of genes related to cytoskeletal architecture, adhesion, and mechanosensing. To capture both differences between WT and KO cells and flow-induced responses, transcript levels were analyzed under static and flow conditions. Direct WT-KO comparisons for each condition are shown in Suppl. Fig. S5. While the relative expression patterns of these genes were largely preserved between WT and KO cells, exposure to shear stress elicited a more pronounced transcriptional response in KO cells, as reflected by larger flow-induced fold changes compared to WT.

**Fig. 7:**
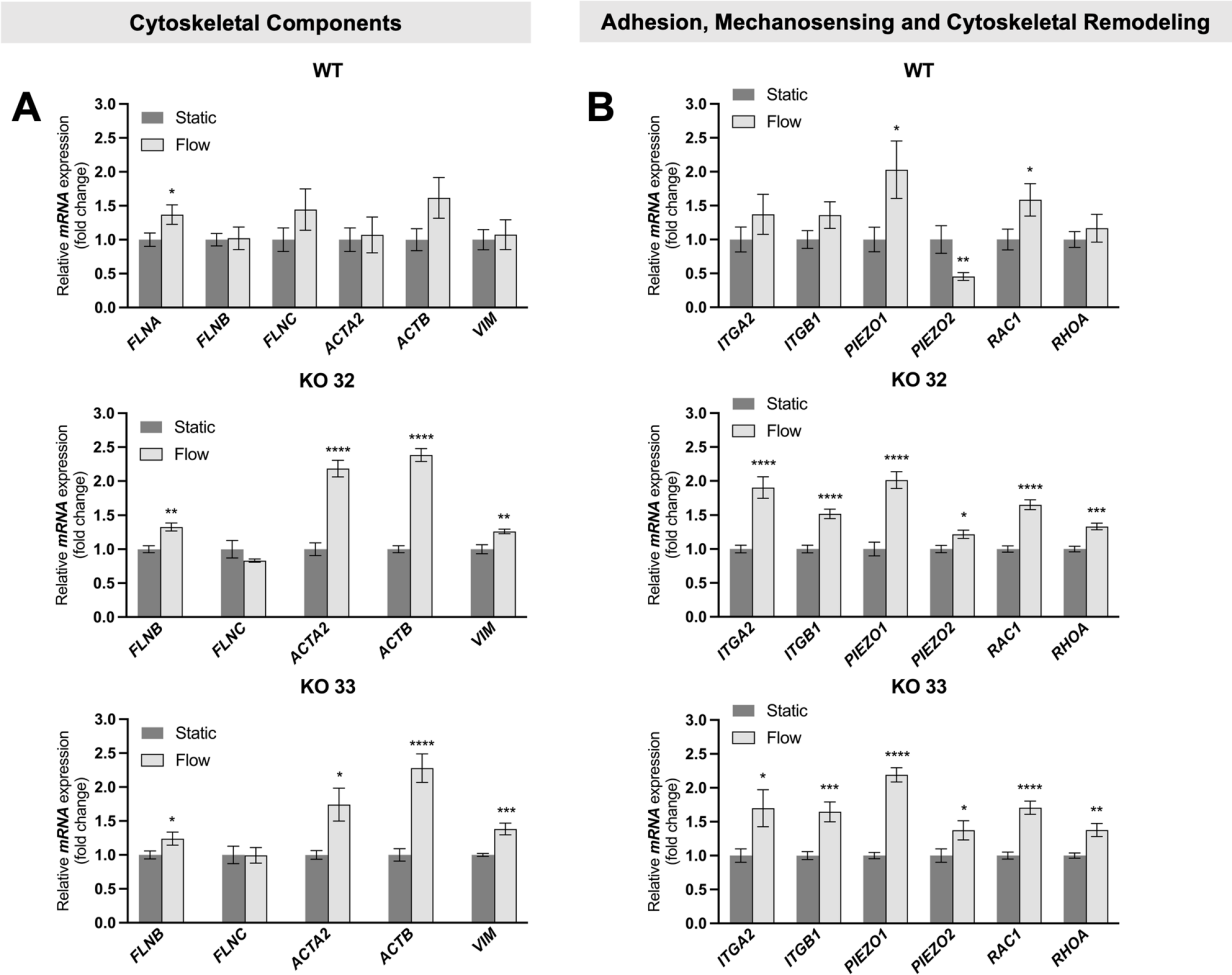
Transcriptional response to laminar flow shear stress. **A** Quantitative PCR (qPCR) revealed differential regulation of filamins and related cytoskeletal components following shear stress exposure in WT and FLNA-KO cells; mean ± SEM (n = 8), unpaired t-test (*p < 0.05, **p < 0.01, *** *p* < 0.001, ****p < 0.0001). **B** qPCR revealed differential regulation of genes related to adhesion-, mechanosensing and cytoskeletal remodeling following shear stress exposure after 24 h in WT and FLNA-KO cells; mean ± SEM (n = 8), unpaired t-test or Mann–Whitney test in case when no normal distribution could be assumed (*p < 0.05, **p < 0.01, *** *p* < 0.001, ****p < 0.0001)

In KO cells, transcripts of a set of cytoskeletal genes—including *FLNB*, alpha-smooth muscle actin (*ACTA2*), *ACTB* and vimentin (*VIM*), but not *FLNC*, were significantly upregulated under high shear stress conditions. In contrast, WT cells showed a significant increase only in *FLNA* transcript levels (Fig. 7A). Among genes associated with mechanosensing and cytoskeletal remodeling, piezo-type mechanosensitive ion channel component 1 (*PIEZO1*) and Rac family small GTPase 1 (*RAC1*)*,* were significantly upregulated under flow, independent of FLNA expression. Of note, KO cells showed a unique upregulation of integrin alpha 2 (*ITGA2*), integrin beta 1 (*ITGB1*) and ras homolog family member A (*RHOA*) while the WT cells exhibited no significant changes in these genes, but a unique downregulation of piezo-type mechanosensitive ion channel component 2 (*PIEZO2*) mRNA (Fig. 7B).

## Discussion

Many roles of the actin-crosslinking protein FLNA have been described, some of which depend on the cell type examined. In tumor cells, however, its functions are controversial and, in part, contradictory [2, 12, 13]. Expression levels in bladder, prostate and colorectal cancers were reported to be low, while in cases of melanoma, glioblastoma and breast cancers, high FLNA expressions were described [14]. For GCTs, neither expression data of, nor functional insights into FLNA were available. We recently described FLNA expression and regulation in GC and KGN cells, a common cell model for human proliferative GC and adult-type human GCTs [4, 5, 8, 9]. In the present study, we generated FLNA-KO clones, and report severe consequences upon deletion of this protein. The proteomic analysis indicated massive cellular changes, which were reflected by an altered abundance of proteins involved in DNA replication, regulation of cell division and cell cycle, cytoskeletal organization and lipid metabolism. In part, the results are in line with data obtained in other cell types but also provide new insights into the further functions of FLNA in KGN cells.

Uncontrolled proliferation and enhanced migratory capacity are hallmarks of tumor cells, and our findings suggest that FLNA contributes to both processes in KGN cells. FLNA deletion led to a pronounced reduction of proliferation rate. This is supported by a decreased expression of PCNA, a marker of DNA replication and S-phase progression. Previous studies have linked FLNA to cell cycle progression and division [27–29]. Consistently, our cell cycle analysis revealed an arrest in the G0/G1 phase and a reduced proportion of cells in the G2/M phase in KO cells, indicating a delay in early cell cycle progression. This shift in cell cycle distribution is also consistent with smaller nuclear size observed in FLNA-KO cells. These findings were substantiated by qPCR and proteomic analyses showing decreased expression levels of distinct key cell cycle regulators, including CDK4 (G1 and G1/S transition), CDK2 (G1/S transition and S phase progression), and CDK1 with cyclin B1 (G2/M transition and mitosis) (reviewed in [30]). Of note, successful post-mitotic daughter cell separation requires a proper actin cytoskeleton remodeling through CDK1-mediated phosphorylation of FLNA [31, 32]. Moreover, substantial evidence indicates CDKs and cyclins are closely intertwined with cell migration and adhesion, as well as invasion in different types of cancer cells [33–36], including ovarian cancer [37]. In breast cancer it was reported that the regulation of migration and invasion depends on FLNA phosphorylation by CDK4/cyclin D1 [38]. Additionally, FLNA has been implicated in cell migration and invasiveness across a variety of cells, tissues and cancers, e.g. in human cortical neurons [39, 40], the vascular system [41, 42], as well as in breast [43], colon [44], papillary thyroid [45], and cervical cancer [46]. The reduced proliferation rate and motility observed in KGN FLNA-KO cells may indicate a less aggressive phenotype in the context of cancer. Interestingly, this phenotype may be associated with alterations in progesterone production. However, this possibility warrants further investigation.

FLNA binds integrins, which are crucial for cell adhesion and migration, and FLNA-deficiency causes an altered integrin expression as our proteomic analysis showed. Specifically, this included downregulation of ITGA2 and ITGA11, and upregulation of ITGA5 and ITGAV, which are discussed in the context of cell proliferation and survival [47–49], as well as migration and reorganization of the actin cytoskeleton [50–53], respectively. The disruption in the FLNA-integrin axis likely results in the differential expression of integrins observed in FLNA-deficient cells, impacting cellular adhesion, migration, and potentially cell structure. Further investigations, however, are needed to address this topic in more detail.

Responses to shear stress have been extensively studied in endothelial cells [54], but not in GCTs. In the present study, perpendicular alignment of KGN cells under shear stress occurred only when FLNA was present. This finding supports an essential role of FLNA in cytoskeletal dynamics, particularly in mechanosensing and in triggering a cellular response to mechanical cues. In support, the enhanced change in transcriptional response of cytoskeletal, adhesion, and mechanosensitive genes under mechanical stress in FLNA-deficient KGN cells, could indicate a compensatory mechanism to mitigate mechanical stress and to retain cellular structure.

It should be noted, that shear stress is a relevant physical force that metastatic tumor cells encounter in the bloodstream or in the lymphatic system [55]. Under such conditions, FLNA may ensure cytoskeletal dynamics required for survival, transit, and colonization at distant sites. Hence, the presence of weak FLNA staining in a subset of GCT samples combined with our functional studies and cell biological analyses in KGN cells, lead us to speculate that tumors with no / low FLNA expression represent a less aggressive subgroup. However, in the patient cohort analyzed, no correlation between FLNA expression and clinical parameters such as Ki67, tumor recurrence or tumor stage was observed. This may be due to the restricted numbers of cases in certain subgroups and incomplete clinical annotation. Substantially larger, well-annotated cohorts will be needed to further assess the potential relevance of FLNA expression in GCTs.

Of note, mechanical cues also play a critical role in ovarian folliculogenesis [56–58]. A transcriptomic study by Zhang et al*.* reported increased expression of *FLNA* in large antral and preovulatory follicles [59], suggesting a possible role for FLNA in advanced follicle stages. Our experimental setup provides a controlled *in vitro* model of mechanical pressure GC cope *in vivo* and offers insights into a potential functional involvement of FLNA.

## Conclusions

Our findings reveal novel functions of FLNA in shaping cellular architecture and regulating key processes such as proliferation, motility and mechanotransduction in KGN cells. They also suggest that FLNA levels may influence tumor behavior. Overall, these data support an essential role for FLNA in GCs under physiological and potentially also under pathological conditions.

## Supplementary Information

Supplementary Material 1

Supplementary Material 2

Supplementary Material 3

Supplementary Material 4

Supplementary Material 5

Supplementary Material 6

Supplementary Material 7

Supplementary Material 8

Supplementary Material 9

## Data Availability

The mass spectrometry proteomics data have been deposited to the ProteomeXchange consortium via the PRIDE partner repository (https://www.proteomexchange.org/) with the dataset identifier: PXD064136.

## Abbreviations

°C: Degree Celsius
ACTA2: α-Smooth muscle actin
ACTB: β-Actin
ANOVA: Analysis of variance
CDK1: Cyclin-dependent kinase 1
CDK2: Cyclin-dependent kinase 2
CDK4: Cyclin-dependent kinase 4
CO_2_: Carbon dioxide
CRISPR/Cas9: Clustered regularly interspaced short palindromic repeats/CRISPR-associated protein 9
DMEM/F12: Dulbecco’s modified eagle’s emedium/nutrient mixture F-12
DNA: Deoxyribonucleic acid
FCS: Fetal calf serum
FLNA: Filamin A
FLNB: Filamin B
FLNC: Filamin C
*g*: Earth’s gravitational acceleration/Relative Centrifugal Force
GC: Granulosa cell
GCT: Granulosa cell tumor
h: Hour
ITGA11: Integrin subunit alpha 11
ITGA2: Integrin subunit alpha 2
ITGA5: Integrin subunit alpha 5
ITGAV: Integrin subunit alpha V
ITGB1: Integrin subunit beta 1
IVF: *In vitro* Fertilization
KO: Knockout
LC–MS/MS: Liquid chromatography–tandem mass spectrometry
min: Minute
mL: Milliliter
mM: Millimolar
mRNA: Messenger ribonucleic acid
MS: Mass spectrometry
NH_4_HCO_3_: Ammonium bicarbonate
nL: Nanoliter
nm: Nanometer
PBS: Phosphate-buffered saline
PCNA: Proliferating cell nuclear antigen
PCR: Polymerase chain reaction
PIEZO1: Piezo-type mechanosensitive ion channel component 1
PIEZO2: Piezo-type mechanosensitive ion channel component 2
qPCR: Quantitative polymerase chain reaction
RAC1: Ras-related C3 botulinum toxin substrate 1
RHOA: Ras homolog family member A
RIPA: Radioimmunoprecipitation assay
RNA: Ribonucleic acid
RT: Room temperature
RT-PCR: Reverse transcription polymerase chain reaction
s: Second
sgRNA: Single guide ribonucleic acid
TMA: Tissue microarray
VIM: Vimentin
WT: Wildtype
µg: Microgram
µL: Microliter
µm: Micrometer
