# Supplementary material for: Knockout of filamin A in KGN granulosa tumor cells impairs proliferation, cell cycle progression, migration, and cytoskeletal organization under mechanical stress
Source: Biol Res. 2026 May 20;59:32. doi: 10.1186/s40659-026-00686-x (PMC13200416; doi:10.1186/s40659-026-00686-x)
Supplement: Supplementary file 1 — Supplementary Material 1 [file 40659_2026_686_MOESM1_ESM.docx]

**Supplementary information**

**Fig. S1: FLNA expression and clinical characteristics in granulosa cell tumors (GCTs)**

(**A**) Original agarose gel image with *FLNA* qPCR product from 4 individual GCT samples with bands sized 105 bp. No reverse transcriptase (-RT) and non-template (H_2_O instead of cDNA) controls were negative. Bands shown in Figure 1B are boxed in black.

(**B**) Clinical characteristics of GCT tissue microarray samples stratified by FLNA immunostaining intensity (n = 51; strong, moderate, weak), including Ki67 proliferation index (n = 51), tumor status (primary versus recurrent; n = 37), and tumor stage (n = 34). Values represent the percentage distribution within each FLNA category.

**Fig. S2: Verification of FLNA-knockout (KO) and expression of FLNB in WT and FLNA-KO KGN cells**

(**A**) Original agarose gel image with FLNA genotyping PCR product bands sized ~1000 bp in WT and ~800 bp in FLNA-KO cells (KO 32, KO 33, KO 35, KO 41) (deletion of 227 bp), respectively. Non-template (H_2_O instead of DNA) control was negative. Bands shown in Figure 2A are boxed in black.

(**B**) Original Western blot images of WT and FLNA-KO KGN cells (KO 32, KO 33, KO 35, KO 41) with FLNA and ACTB bands. Bands shown in Figure 2B are boxed in black.

(**C**) DNA sequence analysis to verify FLNA-KO in KGN cells. The desired deletion at ChrX:153.594.947–153.595.173 in the FLNA gene was detected in all four KO clones (KO 32, KO 33, KO 35 and KO 41) by sequencing, confirming successful gene editing.

(**D**) Western blot images of FLNB expression in WT and FLNA-KO KGN cells.

(**E**) Western blot analysis revealed no significant change of FLNB levels in FLNA-KO clones (KO 32 and KO 33 are shown). ACTB served as a loading control. One-way ANOVA indicated no significant changes (ns, p > 0.05).

**Fig. S3: Proteomic analysis of FLNA-KO cells**

“Cellular Component” subset of the GO analysis of proteins with decreased (**A**) or increased abundance (**B**). The size of each bubble represents the number of genes associated with each term, and the color intensity indicates false discovery rate (FDR).

(**C**) Mass spectrometry-based quantification of PCNA protein levels in WT KGN cells and FLNA-KO clones (KO 32, KO 33, KO 35 and KO 41). PCNA abundance is significantly reduced in all FLNA-KO clones, consistent with impaired proliferation and supporting qPCR data. Statistical analysis was performed using a one-way ANOVA (**** p < 0.0001)

**Fig. S4: FLNA-knockout (KO) alters nuclear size of and progesterone production in KGN cells**

(**A**) Nuclear size in KGN cells was measured by imaging flow cytometry. Data are shown as relative values normalized to WT cells. FLNA-KO cells showed a significant reduction in nuclear size. Data represent mean ± SEM from three independent measurements (n = 3), with each point representing the mean nuclear size per replicate (2×10^4^ cells). Statistical analysis was determined by a one-way ANOVA (* p < 0.05, *** p < 0.001).

**(B)** Steroid production in KGN cells was measured by liquid chromatography-tandem mass spectrometry using supernatants derived from WT and FLNA-KO KGN cells (KO 32 and KO 33) and normalized to corresponding amount of protein. Pregnenolone levels did not differ significantly between WT and FLNA-KO cells. Progesterone was significantly increased in FLNA-KO clone 32, while FLNA-KO clone 33 showed a similar, non-significant trend. Data are presented as mean ± SEM (n = 7 or 3). Statistical analysis was performed using a one-way ANOVA (ns p > 0.05, * p < 0.05, ** p < 0.01).

**Fig. S5: Relative gene expression in WT and FLNA-KO KGN cells under static and flow conditions.**

Relative mRNA expression levels of cytoskeletal, adhesion-related, and mechano-responsive genes analyzed by quantitative PCR. For a duration of 24 h, both WT and FLNA-KO cells were subjected to either static conditions (**A**) or high laminar shear stress (25 dyn/cm²) (**B**). Expression was normalized to the respective housekeeping gene and relative to WT cells under static or flow condition. Data are shown as mean ± SEM (n = 8). Statistical analysis was performed using one-way ANOVA (*p < 0.05, **p < 0.01, ***p < 0.001, ****p < 0.0001).

**Supplementary Table 1: List of oligonucleotide primers used for PCR**

| Gene | Sequence (5´- 3´) | Amplicon size (bp) |
| --- | --- | --- |
| *ACTA2* | F: ACAATGAGCTTCGTGTTGCC | 90 |
|  | R: GAGTCATTTTCTCCCGGTTGG |  |
| *ACTB* | F: ATAGCACAGCCTGGATAGCAACGTAC | 158 |
|  | R: CACCTTCTACAATGAGCTGCGTGTG |  |
| *CDK1* | F: AGCCTAGCATCCCATGTCAA | 106 |
|  | R: TCAGTGCCATTTTGCCAGAA |  |
| *CDK2* | F: TGAAGATGGACGGAGCTTGT | 121 |
|  | R: AAGATGGGGTACTGGCTTGG |  |
| *CDK4* | F: CTTCCCATCAGCACAGTTCG | 121 |
|  | R: CCTTGATCTCCCGGTCAGTT |  |
| *FLNA* | F: CACAGTAACCTGTCCCCAGA | 1031 |
|  | R: CACCTGTGACTTATCCACGGA |  |
| *FLNA* | F: CATTCAGATTGGGGAGGAGA | 105 |
|  | R: ACATCCACCTCTGAGCCATC |  |
| *FLNB* | F: GCCTGTGGATAATGCACGAGA | 219 |
|  | R: GGCTCGATTCCTCTGCCATA |  |
| *FLNC* | F: TATGGCGGTGATGAGATCCC | 93 |
|  | R: TCCAATGGACACTGTGACGA |  |
| *ITGA2* | F: GTCTGTTGCCTGCGATGTAG | 118 |
|  | R: TTGGAAACTGAGAGACGCCT |  |
| *ITGB1* | F: TGCGAGTGTGGTGTCTGTAA | 120 |
|  | R: GAAGGCTCTGCACTGAACAC |  |
| *L19* | F: AGGCACATGGGCATAGGTAA | 199 |
|  | R: CCATGAGAATCCGCTTGTTT |  |
| *PCNA* | F: CAAGTAATGTCGATAAAGAGGAGG | 126 |
|  | R: GTGTCACCGTTGAAGAGAGTGG |  |
| *PIEZO1* | F: TCCCTATCAGACGACCAGGT | 91 |
|  | R: ACAGGGCGAAGTAGATGC |  |
| *PIEZO2* | F: TCCTCCTGCTGCAAAGAAGA | 98 |
|  | R:TTCAGCTCCTCTTGATGCCA |  |
| *RAC1* | F: ATGGAAAACCGGTGAATCTG | 124 |
|  | R: GCAGGACTCACAAGGGAAAA |  |
| *RHOA* | F: GGTGGATGGAAAGCAGGTAG | 124 |
|  | R: GCTGTCGATGGAAAAACACA |  |
| *VIM* | F: AGACAGGTGCAGTCCCTCAC | 104 |
|  | R: GCTTCAACGGCAAAGTTCTC |  |

**Supplementary Table 2: List of all differentially abundant proteins in FLNA-knockout cells.**

**Supplementary Table 3: DAVID analysis of significantly regulated biological pathways.**

**Supplementary Video 1: Time-lapse recording (72 h) of wound-healing scratch assay in WT cells.**

**Supplementary Video 2: Time-lapse recording (72 h) of wound-healing scratch assay in FLNA-knockout cells (KO 32).**

**Supplementary Video 3: Time-lapse recording (72 h) of wound-healing scratch assay in FLNA-knockout cells (KO 33).**

**Supplementary Video 4: Time-lapse recording (48 h) of random motility in WT cells.**

**Supplementary Video 5: Time-lapse recording (48 h) of random motility in FLNA-knockout cells (KO 32).**

**Supplementary Video 6: Time-lapse recording (48 h) of random motility in FLNA-knockout cells (KO 33).**
